# Can carbon finance promote high quality economic development: evidence from China^☆^^☆☆^^☆☆☆^

**DOI:** 10.1016/j.heliyon.2023.e22698

**Published:** 2023-11-25

**Authors:** Lili Jiang, Huawei Niu, Yufan Ru, Aihua Tong, Yifeng Wang

**Affiliations:** a China University of Mining and Technology, Xuzhou, 221116, China; b Suqian University, Suqian, 223800, China; c Fujian Agriculture and Forestry University, Fuzhou, 350000, China

**Keywords:** High-quality economic development, Carbon finance, Heterogeneity analysis

## Abstract

Utilizing provincial panel data from China spanning 2000 to 2020, this study constructed an indicator system for high-quality economic development by incorporating 35 indicators. These indicators stem from five domains: innovative, coordinated, green, open, and shared development. To assess carbon finance development, an evaluation system was created using twelve basic indicators derived from three categories: financial environment, energy efficiency, and scientific and technological progression. The entropy weight method facilitated the computation of indices such as the economic quality development, innovative development, coordinated development, green development, open development, shared development, and carbon finance development indices. Both static and dynamic panel models served to empirically ascertain the specific influence of carbon finance on high-quality economic development. Additionally, regional and temporal variances in carbon finance's impact on high-quality economic development were scrutinized. Findings indicate that provinces like Beijing, Shanghai, Jiangsu, and Guangdong, known for advanced economic development, exhibit elevated levels of high-quality economic and carbon finance development. Nonetheless, disparities among provinces are evident. While carbon finance significantly bolsters China's high-quality economic growth, its influence is not uniformly observed across all five development domains. It primarily augments coordinated, green, and shared development. Furthermore, the role of carbon finance in boosting China's high-quality economic development exhibits regional and temporal variations. The promotion of high-quality economic growth can be achieved by fostering an amenable carbon finance environment, addressing aging, maximizing governmental influence, enhancing transportation infrastructure, propelling new urbanization, and refining education standards.

## 1 Introduction

At present, China's economy is experiencing a high-quality development phase with great growth speed and is in an important period of reforming economic development, the domestic structure of the economy is being optimized, and the development momentum is being transformed. Improving the quality of economic development in various regions has become an urgent problem. The emergence of the low-carbon economy provides a possible way to solve this problem. At present, China's carbon financial market system has made major breakthroughs, and the pricing mechanism is increasingly perfect. According to survey data from the Research Bureau of the People's Bank of China, as of July 2021, the national carbon emissions quota (CEA) had a total of 4.3968 million tons, with a cumulative transaction value of 226 million yuan, which has become the largest market in the world. However, China's carbon finance is still facing the challenge of a lack of depth and breadth of development. However, China's carbon finance still faces the challenge of insufficient development depth and breadth. The establishment of an evaluation system of carbon finance indicators to measure the development level of carbon finance in 31 provinces, autonomous regions and municipalities directly under the Central Government of China and the differences in development between regions is conducive to better analysis of the development level of carbon finance.

China's economy is still large and weak, and the quality of economic development is not high. At present, China is stepping into a historic new era from rich to strong. In terms of economy, it is “from the stage of rapid growth to the stage of high-quality development”. The establishment of a high-quality development evaluation index system to measure the high-quality development level of the economy of 31 provinces, autonomous regions and municipalities in China and the differences in development between regions is worth in-depth discussion. Carbon finance is a financial solution to climate change, a low-cost way to achieve sustainable development, mitigate and adapt to climate change, and a core economic means of low-carbon development. Is the development of carbon finance really conducive to promoting high-quality economic development? Is there heterogeneity in promoting high-quality economic development with carbon finance? These problems are worthy of in-depth study by scholars.

Economic development of high quality prioritizes the comprehensive satisfaction of the populace's escalating demands for an enhanced quality of life. This development embodies a vision characterized by innovation, coordination, environmental consciousness, openness, and collective growth. Such development ensures minimal input of production factors, optimal resource allocation efficiency, reduced resource and environmental expenses, and significant economic and social returns. It is characterized by a persistent enhancement in the quality of goods and services. The improvement in both input-output efficiency and economic efficacy is evident. In this paradigm, innovation emerges as the primary impetus, and ecologically sustainable practices become standard. Additionally, pivotal economic relationships are harmonized, fostering fluid cycles, further deepening reforms, openness in developmental approaches, and emphasizing shared benefits as the foundational objective.

Carbon finance encompasses the allocation of financial capital to bolster environmental rights and interests. Backed by legal frameworks, it employs financial instruments and methodologies to facilitate the trading or circulation of pertinent carbon financial products and derivatives on market-driven platforms. The overarching objective remains the realization of low-carbon, green, and sustainable development.

Low-carbon, green, and sustainable development represents not only the contemporary global trend but also the populace's pressing aspiration for an enhanced quality of life in this new era. Such development additionally serves as an intrinsic prerequisite for enduring economic and social progression, thereby signifying a hallmark of high-quality development. The accelerated advancement of green, low-carbon technologies, combined with the notable augmentation of economic capabilities, has facilitated the expedited rehabilitation of compromised ecological environments. It becomes imperative to further embed the principles of low-carbon, green, and sustainable development. Such integration necessitates the swift establishment of policy direction, institutional frameworks, and legislative measures to foster carbon finance. This, in turn, will stimulate the growth of sectors like energy conservation, environmental protection, clean production, and clean energy. Ultimately, it aims to enhance the green, low-carbon, and cyclical dynamics of the economic system, paving the way for the achievement of superior economic development.

Drawing from the current research landscape of carbon finance and high-quality economic development, this study primarily focuses on three analytical dimensions: The prevailing developmental state of high-quality economic advancement and carbon finance across various Chinese regions. The potential of carbon finance as a catalyst for high-quality economic growth. The spatial and temporal heterogeneity of carbon finance's influence on high-quality economic progression.

For detailed analysis, this study employs the entropy weight method to quantify the high-quality economic development index and the carbon finance development index. Subsequent empirical analyses leverage static panel models, dynamic panel models, and spatial autocorrelation tests to scrutinize the influence of carbon finance on high-quality economic development, along with its temporal and regional variations.

In juxtaposition with extant research, this study offers several key contributions: **Indicator Research**: This study endeavors to mitigate the shortcomings present in comprehensive measurement indices of high-quality economic development found in prior research. Taking into account the availability of indicators, this work establishes 12 secondary and 35 tertiary indicators across five dimensions—innovation, coordination, green initiatives, openness, and sharing. Consequently, a comprehensive index system measuring high-quality economic development is constructed. Furthermore, the assembly of a carbon finance development index system remains nascent in the literature. Drawing from three principal arenas—financial environment, energy efficiency, and technological progress—this study introduces 12 fundamental indicators to devise an evaluative system for carbon finance development. This substantially augments the body of work focused on carbon finance index system construction. **Research Perspective**: While a plethora of research evaluates the influence of finance on high-quality economic development and its overarching index, scant attention has been dedicated to measuring specific sub-indices. Concurrently, there remains a dearth of studies quantifying the carbon finance development index. This investigation calculates the carbon finance development index, targeting high-quality economic development, to discern the impact of carbon finance therein. It proposes an innovative trajectory advocating high-quality economic development through carbon finance augmentation, further enriching academic discourse on the subject. **Research Content**: This manuscript delves into the trajectory, regional discrepancies, and temporal variations in the effect of carbon finance on high-quality economic development. In doing so, it broadens the scope of research bridging high-quality economic development and carbon finance. **Practical Implications**: Beyond academic contributions, this study furnishes empirical evidence bolstering the advancement of both high-quality economic development and carbon finance. It lends empirical backing to the proposition that carbon finance can amplify high-quality economic development, underscoring its pivotal role in endorsing a sustainable low-carbon pathway. As the developmental paradigm shifts towards “coordinated enhancement of carbon reduction, pollution mitigation, green proliferation, and growth,” carbon finance continually refines its essence. Transitioning from exclusively backing green initiatives like pollution control and climate action, it now accentuates “dual control” carbon emission initiatives, gradually steering towards encompassing zero carbon finance, inclusive of transition finance.

The organizational structure of this article unfolds as follows: Introduction: This section delineates the research background and significance, addresses the core questions tackled, and highlights the potential contributions of the article. Literature Review: An assessment of pertinent existing literature is presented. Theoretical Framework: This segment establishes the foundational theories underpinning the study. Variable Selection and Modeling: This part elucidates the choices of variables and the modeling approach adopted. Empirical Analysis: Empirical research findings are presented and discussed in detail. Conclusion and Policy Recommendations: The article culminates with a summary of findings and pertinent policy suggestions.

## 2 Literature review

### 2.1 High-quality economic development

Research on the connotation of high-quality economic development includes the following aspects [1] argues that the connotation of the quality of economic growth should take into account the regional financial development, the improvement of education quality and the effect of environmental governance in Economic Growth of Various Countries. Soviet scholar B.A. Kalma (1983)^[^^2^^]^ points out that the essence of economic growth covers the relationship between speed and quality. When economic development has a certain scale, we should pay attention to qualitative improvement at the same time. Dornbusch and Fischer (1997) ^[^^3^^]^points out that the improvement of economic growth quality mainly comes from the improvement of resource utilization efficiency and factor productivity. According to Thomas (2000)^[^^4^^]^, equitable distribution of opportunities, sustainable environment, global risk management and governance structure are important meanings of the quality of economic growth. Mlachila (2014) ^[^^5^^]^believes that the high-quality growth of developing countries is stable, strong and sustainable, and can promote the harmonious and friendly development of society.

The studies regarding the measurement of economic development with high-quality: The existing literature not only measures high-quality development with a single index, but also measures it with a multi-dimensional index system. In terms of a single indicator, Jorgenson and Griliches (1967)^[^^6^^]^ all take total factor productivity as the source of economic growth in economic models. Saleem et al. (2019)^[^^7^^]^ believes that TFP is the driving factor behind economic growth, and they use the Cobb-Douglas production function to evaluate TFP. More scholars evaluated by building a comprehensive index system [2].^[^^8^^]^ evaluates the economic development quality of Russian mining industry by establishing a spatial qualitative evaluation index system of resource-based regional economy including 16 s-level indicators from three dimensions, namely agglomeration and resource distribution density, transportation information infrastructure development, and technical conditions of economic sectors [3]. ^[^^9^^]^considers the average annual productivity growth rate and per capita development index, and uses the matrix model to measure and evaluate them. Qi (2016) ^[^^10^^]^uses three indexes, namely economic structure, production performance and regional coordination, to construct an index system of high-quality development.

Compared with the measurement for the underpinnings and level of high-quality economic development, it is more important to study the key factors of high-quality economic development to enhancing the level of high-quality economic development. Some scholars have explored the influencing factors of high-quality economic development from multiple perspectives [4].^[^^11^^]^ believes that the quality of Italian economic development should be enhanced by enhancing the sense of belonging of the public, increasing social capital and regulating public expenditure [5]. ^[^^12^^]^discusses the positive effect of knowledge-based economic model on the quality of economic development. Using the panel model and system GMM model, Rongrong Wei, Ying Xia, and Zhaopeng Yu (2020) ^[^^13^^]^analyze the relationship between high-quality economic development, technological innovation, and financial development and conclude that technological innovation and financial development are conducive to high-quality economic development. Wentao Gu, Jiaye Wang, Xiyuan Hua, and Zhongdi Liu (2021)^[^^14^^]^ note that high-quality development is now a must for sustainable development. Using the spatial lag model [6], ^[^^15^^]^analyze the interaction between environmental regulation, foreign direct investment, and high-quality economic development, and conclude that environmental regulation is advantageous for promoting high-quality economic development.

### 2.2 Carbon finance

The research on carbon finance mainly includes the following aspects. In the context of sustainable development, scholars' research on carbon finance mainly focuses on four aspects. First, regarding carbon financial transaction prices [7], ^[^^16^^]^ and others pointed out that the carbon finance transaction price is essentially the internalization of the external cost of carbon emission control unit, and the effective operation of the price is an important prerequisite for the carbon market to achieve environmental and economic benefits. Second, regarding the role of financial institutions in the development of carbon finance, Hugh [8] ^[^^17^^]^believes that financial institutions have a core position in the carbon finance market, carbon banks can store carbon reserves, and carbon banks can be used as a new type of carbon trading in the future. The business organization obtains the corresponding income through the transaction. Malte Schneider, Holger Hendrichs, Volker H. Hoffmann ^[^^18^^]^took 495 institutions as the research object and conducted an in-depth analysis of the impact of carbon credits as an incentive policy on the industrial chain, fully recognized the contribution of carbon bank. Third, regarding the influencing factors of carbon finance, Labatt Sonia, White Rodney (2018) ^[^^19^^]^pointed out that the energy chain, energy-intensive industries, and climate change are important influencing factors for the development of carbon finance. Fourth, regarding the measurement of the development level of carbon finance [9], ^[^^20^^]^used the time-series multi-index model to determine the index weights and calculated the 2014–2019 China provincial carbon finance development index, noting that China's overall carbon finance development level showed an upward trend, and the number of CDM projects was on the rise. The number of new energy buses in operation, the completion of investment in industrial pollution control, and urban afforestation coverage promote the development of carbon finance, while the urban registered unemployment rate and industrial added value inhibit the development of carbon finance.

The research on the carbon financial market mainly includes the following aspects. Ding,G and Deng, YL (2019)^[^^21^^]^ take Fujian Province, China as an example and find that in the process of establishing the carbon emission trading market, government departments should strengthen policy support for talent training, carbon credit evaluation system construction and carbon finance projects [10]. ^[^^22^^]^believe that regional carbon market will be the development trend of carbon finance market in the future, and cooperation between countries will be the mainstream of using carbon market to solve global climate change [11]. ^[^^23^^]^and other scholars put forward a new and efficient carbon financial market trading system mechanism, which is more in line with the real environment of the European Union [12].^[^^24^^]^ conduct an in-depth analysis of the volatility of the carbon price in the European Union and believe that various policies launched by the financial market restricted investors in the market to make profits by futures trading, resulting in significant volatility of carbon futures price and contagion to the carbon spot market. Anupam [13] ^[^^25^^]^uses the bivariate DCC-GARCH model to study the impact of European carbon finance market risks on other real economic markets, and finds that the risks of carbon finance market would be significantly transmitted to other traditional real markets through the supply chain, and the fluctuating carbon price would lead to the uncertainty of the price index of relevant industries [14]. ^[^^26^^]^uses the CopulA-CO ES model to measure the risk spillover effect between carbon markets, and the empirical results show that, compared with the traditional value at risk, the conditional value at risk can better measure the volatility risk of carbon market.

### 2.3 Impact of carbon finance on high-quality economic development

Some academics have studied how carbon emissions affect economic growth, but there is a dearth of literature on the effects of carbon finance on robust expansion [15].^[^^27^^]^ proposes that expanding carbon financing in China's construction industry is an economic means to reduce carbon emissions, and appropriate policies and economic means should be selected to reduce carbon emissions [16].^[^^28^^]^found a positive correlation between air pollution, energy consumption and water resources [17].^[^^29^^]^ took Japan as an example and analyzed the relationship between energy and income with the environmental Kuznets curve, and found out how energy changes. The contribution rates of consumption, export and import to a country's economic growth are 33.55 %, 1.027 % and 7.126 % respectively. Analyses revealed that CO₂ emissions accounted for 16.24 %, 23.89 %, and 44.18 %, respectively [18]. ^[^^30^^]^ conducted an empirical examination of the interplay between economic growth, energy consumption, financial development, trade openness, and CO₂ emissions within Nigeria. The research identified that financial development heightened energy demand while concurrently decreasing CO₂ emissions. Intriguingly, economic growth diminished energy consumption but amplified CO₂ emissions. The study advocates substantial investments in Nigeria's financial sector to propel these domains towards adopting efficient, sustainable renewable energy solutions. Subsequent work by Ref. [19] ^[^^31^^]^ determined that financial development bolstered energy demand in South Africa. Furthermore, affluence levels and trade openness exhibited positive associations with energy consumption, consequently escalating it. Another study by Ref. [20] ^[^^32^^]^ discerned that the composite variables of globalization, energy consumption, and economic growth wielded dynamic impacts on South Africa's environment. Notably, 7.96 % of energy utilization and 0.80 % of globalization were potentially causative of corresponding environmental degradation, with 72.52 % and 1.39 % respectively. The research underscored the imperative for judicious energy policies complemented by an uncontaminated energy mix. Lastly [21], ^[^^33^^]^ postulated that in France, economic growth correlates with various factors including electricity consumption, financial evolution, capital, imports, and exports. To perpetuate electricity utilization, an infusion of entrepreneurial innovation is paramount to shape opportunities and stimulate economic advancement. Xiangsheng Dou, Huanying Cui (2016) ^[^^34^^]^pointed out that China is facing climate change, environmental protection and energy security, and believes that China must create a low-carbon society to solve these problems, and China must vigorously promote the development of low-carbon systems, technologies, subsidies and taxes, financing and investment, and lay a solid foundation for the comprehensive development of a low-carbon society. Dmitriy Li1, Jeong Hwan Bae1 and Meenakshi Rishi (2021) ^[^^35^^]^analyzes the relationship between energy, carbon emissions and economic growth in the sub-Saharan region and pointed out that all types of energy aid contribute to economic growth in the long run. Itbar KhanLei and Han Hayat Khan (2022)^[^^36^^]^ examined the link between economic growth and carbon emissions in 2022 and concluded that the deployment of renewable energy enhances quality of the environment by lowering carbon emissions and influencing economic development.

Qiang [22] ^[^^37^^]^used the data of 182 countries to analyze the impact of trade liberalization on carbon emissions decoupling, and the results showed that for high-income and upper-middle-income countries, trade liberalization reduced carbon emissions, while for lower-middle-income countries, trade liberalization had no significant impact on carbon emissions, and for low-income countries, trade liberalization increased carbon emissions. Qiang [23] ^[^^38^^]^used the data of 134 countries to explore and analyze the relationship between urbanization and economic growth as well as economic growth and environmental quality, and the results showed that urbanization is conducive to economic growth, and economic growth has a greater positive impact on the ecological footprint of society than carbon emission. Based on the data of 147 countries [24], ^[^^39^^]^analyzed the impact of structural changes on per capita carbon emissions from the perspectives of energy, trade, society and economy, and found that economic growth and economic structure were the most significant positive and negative factors affecting carbon emissions globally [25]. ^[^^40^^]^analyzed the impact of energy efficiency on carbon emissions by using the data of transportation departments in 30 provinces in China, and the results showed that the inhibition effect of energy efficiency on carbon emissions in the transportation industry increased with the improvement of energy efficiency [26]. ^[^^41^^]^analyzed the impact of renewable energy on economic growth by using nearly 20 years of data from 120 countries, and the results showed that global renewable energy is conducive to promoting economic growth and environmental improvement. With the increase of urbanization rate, the negative impact of renewable energy on economic growth first weakens and then increases，while the positive impact first decreases and then increases [27]. ^[^^42^^]^analyzed the relationship between digital economy and carbon dioxide emissions, and the result showed that the two showed a U-shaped relationship, suggesting that natural resources should be fully considered in the process of digital economy development, while anti-corruption efforts in the field of environmental protection should be strengthened [28].^[^^43^^]^ analyzed the impact of trade on carbon emissions, and the results showed that the impact of trade on carbon emissions is heterogeneous and asymmetrical, with both technical and structural effects.

In terms of high-quality economic development the existing literature has done some research on the connotation, level measurement and impact factors of high-quality economic development, and some scholars have also done some research on sustainable economic development, which provides a certain basis for this study. However, there is no consensus on the connotation of high-quality economic development in the academic community, and the construction of the evaluation index system needs to be further improved. This paper selects 35 basic indicators for analysis to further enrich the measurement system of high-quality economic development. It not only measures the economic high-quality development index, but also further measures the economic innovation development index, the economic coordinated development index, the economic green development index, the economic open development index and the economic sharing development index. In terms of carbon finance, scholars have carried out research on carbon finance transaction price, the role of financial institutions in carbon finance, the impact factors of carbon finance and the measurement of carbon finance development level. In terms of carbon finance market, scholars have conducted relevant research on the operation of carbon emission trading market, the pricing of carbon emission rights and their financial derivatives, and the risk of carbon finance market. However, there are few literatures on the measurement of carbon finance development level as a whole. This paper selects 12 basic indicators to measure the carbon finance development level, providing a new way to study and discuss the carbon financial development level. Scholars have mainly analyzed the relationship between economic growth and environmental quality. There are few documents that specifically analyze the impact of carbon finance development on high-quality economic development. This article specifically analyzes the impact of carbon finance development on high-quality economic development, and on this basis, further studies the path of carbon finance's impact on high-quality economic development. It analyzes the regional and temporal characteristics of carbon finance's impact on high-quality economic development, and provides a favorable supplement to existing research, filling the gap in research on the relationship between carbon finance and high-quality economic growth.

## 3 Theoretical analysis

**Fig. 1:**
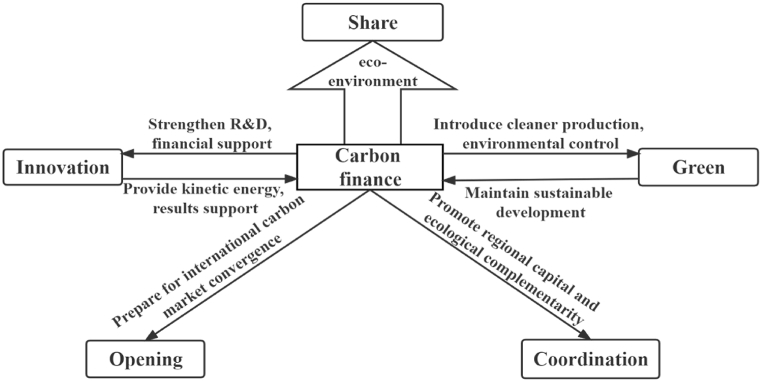
The logic of the impact of carbon finance on high-quality economic development.

High-quality economic development includes the five development dimensions of “innovation, coordination, greenness, openness, and sharing”. Carbon finance promotes high-quality economic development through these five dimensions（Fig. 1）.

In terms of green, carbon finance is the core of the green economy. Through the guiding effect of investment and financing, it encourages the development of a green economy, such as new energy and clean production, and guides the flow of capital from high-polluting and high-emission industries to green industries. At the same time, it promotes environmental regulation, achieves green, healthy and sustainable economic growth and comprehensive environmental improvement, and ultimately feeds back on carbon finance and moves toward a sustainable development path.

In terms of innovation, economic growth that relies on extensive resources, investment, and the environment is unsustainable. New kinetic energy driven by innovation must be tapped. The development of carbon finance can provide financial support for innovative undertakings and help to increase R&D and investment in science and technology, and the transformation of scientific research results is conducive to supporting the real economy related to carbon finance, injecting new impetus into the development of carbon finance.

In terms of coordination, the development of carbon finance improves the ecological value compensation mechanism and implements the principle of whoever emits is responsible, which is conducive to the value of ecological resources in underdeveloped areas. At the same time, carbon finance can help developed regions complete carbon emission tasks, promote green and clean production, and achieve sustainable development.

In terms of openness, against the backdrop of countries around the world responding to climate change, a community with a shared future for human climate has become an inevitable choice. The international carbon market and carbon finance market are becoming increasingly mature. Carbon emission standards may become high barriers to international trade in the future. However, China's carbon market is not yet perfect, and the carbon financial market is still in the preliminary stages of development. To integrate with the foreign carbon trading market, the development of carbon finance is imminent.

In terms of sharing, the development of carbon finance is conducive to strengthening environmental control, promoting all sectors to jointly protect society, the natural environment and resources, and safeguarding the environmental rights and interests of the people. The development of carbon finance pushes China's environmental Kuznets curve to an inflection point, forming a situation in which economic development drives environmental improvement.

In conclusion, carbon financing theoretically encourages the high-quality growth of China's economy, and there are five paths: innovation, coordination, green, openness, and sharing. This paper proposes the following assumptions.

Hypothesis 1 Carbon finance can effectively promote high-quality economic development.

Hypothesis 2 Carbon finance promotes some or all five paths of “innovation, coordination, greenness, openness, and sharing".

## 4 Variables and models

### 4.1 Variables

According to the empirical arrangement, the variables involved in this paper are shown in Table 1.

**Table 1:** Variable selection.

Type	Name	Meaning
Explained variables	HQED	High-quality Economic Development Index
	EID	Economic High Quality Innovation and Development Index
	ECD	Economic High Quality Coordinated Development Index
	EGD	Economic High Quality Green Development Index
	EOD	Economic High Quality Open Development Index
	ESD	Economic High Quality Shared Development Index
Core explanatory variables	CARBON	Carbon Finance Development Index
Control variables	OLD	Elderly dependency ratio
	CHILD	Adolescent dependency ratio
	BIRTH	Birth rate
	SHIYE	Unemployment rate
	ZHF	Government expenditure level
	INF	Infrastructure construction level
	CHZ	Urbanization level
	TIME	Duration of education in higher education
	EDU	Number of educated individuals in higher education

### 4.2 Introduction to variables

#### 4.2.1 Explained variable

This study designates the index of high-quality economic development as the dependent variable. Rooted in the principles of “innovation, coordination, green, openness, and sharing,” which encapsulate the essence of the contemporary developmental paradigm, these guidelines also delineate China's quintessential objectives for securing high-quality economic development in the modern era. Viewing high-quality economic development holistically, this study considers these five sub-objectives as distinct layers within a systemic framework. In adherence to principles of comprehensiveness, representativeness, scientific rigor, comparability, and data accessibility, and building upon research conducted by Rong & Ying (2020), Wen & Wang (2021), and Xiao & Huang (2021), a set of 35 secondary indicators has been curated to constitute the high-quality economic development measurement system. A detailed breakdown is provided in Table 2.

**Table 2:** Evaluation system of high quality economic development (HQED).

Evaluation dimension	Subindices	Basic indicator
Economic High Quality Coordinated Development Index (ECD)	Regional coordination	Regional consumption ratio
		Regional income ratio
	Urban and rural coordination	Urban and rural fixed asset investment ratio
		Urban–rural income ratio
		Urban–rural consumption ratio
	Industrial coordination	Advanced industrial structure
	Investment and consumption coordination	Investment consumption ratio
		Investment rate
		Consumption rate
	Welfare sharing	Per capita medical and health expenditure
		Number of doctors per 10,000 people
		Number of public libraries
		Education expenditure per student
		The number of hospitals and health centers per 10,000 people
		Social Security Expenditure Per Capita
Economic High Quality Shared Development Index (ESD)	Financial sharing	Insurance depth
		Insurance breadth
		Financial depth
		Financial breadth
Economic High Quality Innovation and Development Index (EID)	Innovation input	Investment level of research and development funds
		R&D spending level
	Innovation output	Per capita patent authorizations
		Proportion of transaction volume in the technology market
Economic High Quality Open Development Index (EOD)	Open for investment	The proportion of foreign direct investment
	Open to trade	The proportion of total import and export
Economic High Quality Green Development Index (EGD)	Green life	Environmental spending
		Forest cover rate
		Forest area
		Domestic waste disposal
		Area of nature reserve
		Number of nature reserves
	Green production	Waste water discharge per unit of industrial added value
		Waste discharge per unit of industrial added value
		Sulfur dioxide emissions/total industrial output value
		Coal consumption/regional GDP

**Innovative Development:** The current traditional economic model, characterized by its low efficiency and added value, is inadequate for the demands of high-quality economic development. Positioned as the primary catalyst for high-quality advancement, innovative development necessitates specific infrastructural and environmental foundations. Strategic investments in the domains of science, technology, and education are imperative to actualize the fruition, implementation, and transformation of technological and scientific innovations, thereby providing a continuous impetus for socio-economic growth. Key metrics for innovative development include: the investment levels in research personnel and R&D funds, indicative of innovation input; while the ratio of technology market turnover and the per capita number of granted patents typify innovation output.

**Coordinated Development:** Given the prevailing challenges of imbalanced and insufficient development, achieving high-quality economic development necessitates an emphasis on coordination across various facets including regional disparities, urban-rural divides, industrial sectors, and the interplay between investment and consumption. Efforts should target identifying and rectifying these gaps. The coordinated development framework can be compartmentalized into four criteria: regional coordination, urban-rural integration, industrial coherence, and a balance between investment and consumption. Metrics for regional coordination include the regional consumption ratio and regional income ratio. The disparity in urban and rural development is gauged by the fixed asset investment ratio, disposable income ratio, and the per capita consumption disparity between urban and rural inhabitants. Industrial coordination is assessed by comparing the output value ratios of tertiary to secondary sectors. Lastly, the harmonization between investment and consumption is evaluated using the ratio of investment to consumption, the investment rate, and the consumption rate.

**Green Development:** Reflecting upon historical developmental patterns, it becomes evident that the conventional, unsustainable modes of development have inflicted substantial damage upon the ecological environment. This detriment not only endangers human health but also compromises societal security and stability. One of the tenets of high-quality development, green development, underscores the significance of fostering a symbiotic relationship between humans and nature while ensuring sustainable economic progression. The focus extends beyond the mere preservation of the current ecological environment to include the rehabilitation of previously impacted regions. Under this umbrella, green development bifurcates into two primary criteria: green living and sustainable production. Metrics for green living encompass environmental protection expenditure, forest coverage rate, total forested area, domestic waste management, and both the magnitude and count of protected natural reserves. For sustainable production, indicators like wastewater from added industrial value, waste production, exhaust emissions, and energy consumption per unit of output are taken into account, with these being inversely proportional to green development.

**Open Development:** Since its economic liberalization, China has persistently expanded both the breadth and depth of its external openness. Prioritizing domestic circulation to meet domestic demand has emerged as a pivotal aspect of open development. Key indicators for assessing open development include the ratio of foreign direct investment and the proportion of imports and exports.

**Shared Development:** Central to the concept of high-quality development is the principle of shared development, which mandates a fair and equitable distribution of economic benefits among the populace. To operationalize this, the focus is placed on social public services and resources integral to the well-being of individuals. Accordingly, shared development is bifurcated into two key dimensions: welfare distribution and financial inclusivity. Indicators for welfare distribution include per capita expenditure on healthcare, the number of healthcare facilities, healthcare professionals, public libraries, educational expenditure, and social security spending. Meanwhile, financial inclusivity is gauged through indicators like financial penetration, depth, and the breadth and depth of insurance coverage.

#### 4.2.2 Core explanatory variables

**Carbon Finance:** Carbon finance encompasses the systems facilitating carbon financial trading activities during greenhouse gas emission reduction processes. It also refers to the funds allocated for services supporting these activities. This domain primarily includes carbon emissions and trading, carbon-related financial activities undertaken by financial and service institutions, and corporate low-carbon emission reduction projects. Drawing from the investigations of [29] ^[^44^]^ and Zheng Qunzhe (2022)^[^45^]^ on China's regional carbon finance development, this study formulates the benchmark tier of the carbon finance index system through three dimensions: the financial environment, energy efficiency, and scientific and technological advancement. Indicator selection adheres to principles of validity, representativeness, and feasibility. Indicators gauging the financial environment encompass the financial industry's value-added, carbon emission deposit credit, carbon emission loan credit, and carbon emission insurance credit. Energy efficiency is assessed through metrics such as carbon emission intensity, energy consumption intensity, electricity consumption intensity, and natural gas consumption intensity. The proportion of R&D personnel, number of R&D institutions, investment in science and technology, and R&D expenditure serve as indicators to evaluate the progress in scientific and technological development. Detailed measurement methodologies are elucidated in Table 3.

**Table 3:** Carbon finance (CF) evaluation index system.

Evaluation dimension	Basic indicator	Evaluation dimension	Basic indicator
Financial environment	The proportion of added value in the financial industry	Energy Efficiency	Carbon intensity
	Carbon emissions/deposit quota		Energy consumption intensity
	Carbon Emissions/Loan Quota		Power consumption intensity
	Carbon Emissions/Insurance Credit		Natural gas consumption intensity
Technological development	Proportion of scientific and technical personnel	Technological development	Government finance technology investment
	Number of research and development institutions		Research and development investment intensity

#### 4.2.3 Control variables

This study incorporates a selection of control variables, encompassing the population dependency ratio (split into elderly and child dependency ratios), population birth rate, unemployment rate, government expenditure, transportation infrastructure development, urbanization rate, and educational attainment (measured both by per capita educational duration and the proportion of individuals receiving higher education). Detailed methodologies for constructing each variable are delineated in Table 1.

The population dependency ratio comprises both the elderly dependency ratio (OLD) and the child dependency ratio (CDR). The shifts in China's population age structure have manifested significant time-varying characteristics in their impacts on the current account of trade and economic growth, exerting both short-term and long-term influences. Notably, the implications of these changes on the current account and economic growth differ. A reduction in the child dependency ratio positively influences the current account, yet concurrently dampens economic growth. Conversely, an uptick in the elderly dependency ratio similarly bolsters the current account while hindering economic growth. These observations suggest that the decrease in the child dependency ratio combined with the increase in the elderly dependency ratio has contributed to a current account surplus while marginally slowing economic growth.

Birth rate (CHILD): Fluctuations in birth rate influence the size, structure, and quality of the population, which in turn have implications for the economic progression of a country or region. An increase in the birth rate positively correlates with high-quality economic development.

Unemployment rate (SHIYE): Elevated unemployment rates tend to decrease consumer spending propensity, thereby influencing production and consumption. This results in a diminished national economic growth rate. A higher unemployment rate is negatively correlated with high-quality economic development.

Government expenditure (ZHF): Increased government spending can catalyze production, augment demand, foster job creation, facilitate wealth redistribution, and incentivize business investments. Thus, government expenditure positively influences high-quality economic development.

Transportation infrastructure development (INF): The primary contribution of enhanced transportation infrastructure to economic development is the reduction in transportation costs and duration. The resultant contraction in time and space dimensions significantly facilitates regional interactions and cooperation. The development of transportation infrastructure is positively associated with high-quality economic growth.

Urbanization rate (CHZ): The rate of urbanization plays a pivotal role in fostering technological innovations, refining social labor divisions, and catalyzing economic expansion. Concurrently, job growth and the benefits of population density resulting from economic development further spur the progression of urbanization. There exists a reciprocal relationship between the urbanization rate and economic growth. An increased urbanization rate has a positive bearing on high-quality economic development.

Educational attainment is predominantly indicated by the duration of formal education (TIME) and the percentage of individuals pursuing higher education (EDU). An enhanced educational system cultivates a workforce characterized by high qualifications and advanced skills, thereby elevating the competence and dexterity of the labor population. Such a workforce aligns well with the demands of economic development, augmenting productivity and fostering innovation. Enhanced educational qualifications correlate with increased workforce productivity, translating to heightened value creation and contributions, which propel economic growth. Besides elevating individual earnings, education amplifies the collective economic output of a nation. Both the duration of formal education and the percentage of higher education enrollees positively influence high-quality economic development.

### 4.3 Entropy weight method

Given that each specific indicator possesses distinct units, standardization is imperative to consolidate the index values within the 0 to 1 range. Subsequent to this normalization, dimension values are ascertained through weighting. The standardization procedure unfolds as（1）and （2）：

(1) $$\text{Positive}\;\text{indicator}：\;B_{ij}=\frac{x_{ij}-m_{ij}}{M_{ij}-m_{ij}}$$

(2) $$\text{Negative}\;\text{indicator}:B_{ij}=\frac{x_{ij}-m_{ij}}{M_{ij}-m_{ij}}$$

Let $B_{ij}$ denote the dimensionless value of the j ^th^ specific index within the i ^*th*^ dimension during index computation. Where $x_{ij}$, $m_{ij}$、and $M_{ij}$ represent the actual, minimum, and maximum values of the j ^*th*^ specific index of the dimension, respectively.

Calculating the entropy of each indicator，For details, please refer to（3）and（4）：

(3) $$e_{j}=-\frac{\sum (f_{ij}\;\ln\;f_{ij})}{\mathrm{LN}\left(n\right)}$$

(4) $$f_{ij}=\frac{B_{ij}}{\sum_{j=1}^{n}B_{ij}}$$

Calculating the entropy weight of the j ^th^ index. For details, please refer to（5）

(5) $$w_{j}=\frac{1-e_{j}}{\sum_{j=1}^{n}\left(1-e_{j}\right)}$$

Calculating the comprehensive weights of indicators $\lambda_{j}$，For details, please refer to（6）

(6) $$\lambda_{j}=B_{ij}w_{j}$$

On the basis of the above analysis, each specific index is calculated.

### 4.4 Model setting

#### 4.4.1 Static panel regression model

This study introduces a static panel regression model. Within the framework of panel data linear regression, when the model's intercept term varies across cross-sections or distinct time series while maintaining consistent slope coefficients, such a model is classified as a fixed-effect model. Beyond the fixed-effect model, established panel data analytical methodologies also encompass the random-effects model. The fixed-effect model operates under the premise that all integrated studies share an identical true effect size. Conversely, the random-effect model posits that the true effect differs among individual studies. Consequently, the computed average of the combined effect size varies depending on the model applied (7).

(7) $$HQED_{t}=\lambda_{i}+\beta_{i}CF_{t}+\upsilon_{i}control_{t}+u_{t}$$

Within (7) the fixed-effect model, $\lambda_{i}，i=1,2,.....,n$ serves as a constant, whereas in the random-effect model, $\lambda_{i}$ functions as a random variable. The variable $HQED_{t}$ acts as the dependent variable, signifying high-quality economic development. $CF_{t}$ denotes the carbon financial development index, while control embodies the control variable.

#### 4.4.2 Dynamic panel regression model

Building upon the static panel data model and accounting for the temporal correlation of the dependent variable, the dynamic panel data model integrates the time-lagged term of the dependent variable. China's 31 provinces and cities exhibit heterogeneous development patterns with pronounced spatial attributes. The dynamic panel model primarily encompasses both the difference GMM and system GMM models, with the latter rectifying certain omissions inherent in the difference GMM. To facilitate a comprehensive analysis, this study employs the system GMM model for further examination. The system GMM inherently incorporates the spatiotemporal term HQEDit−1 of the dependent variable. The specific formulation is delineated (8).

(8) $$HQED_{t}=\tau HQED_{t-1}+\beta CF_{t}+\theta W_{i}control_{t}+u_{i}$$

5. Empirical analysis

### 4.5 Data

This paper selects the panel data of 31 provinces, autonomous regions, and municipalities directly under the Central Government in China from 2000 to 2020 as the research object. The data came from the “China Statistical Yearbook”, “EPS Database”, “China Science and Technology Statistical Yearbook”, etc. Individual missing data was supplemented by trend complementation. The descriptive statistics of the data are shown in Table 4.

**Table 4:** Descriptive statistics of variables.

Variables	Average value	Standard deviation	Minimum value	Maximum value	Variables	Average value	Standard deviation	Minimum value	Maximum value
HQED	0.194	0.106	0.049	0.576	CHILD	24.857	7.288	9.640	44.650
EID	0.097	0.086	0.002	0.574	BIRTH	11.374	3.059	3.660	23.200
ECD	0.490	0.059	0.375	0.690	SHIYE	3.487	0.714	0.760	6.500
EGD	0.511	0.073	0.233	0.732	ZHF	0.237	0.187	0.000	1.679
EOD	0.177	0.163	0.002	0.745	INF	0.647	0.491	0.006	2.173
ESD	0.242	0.098	0.066	0.553	CHZ	50.401	15.726	18.910	89.600
CARBON	0.622	0.090	0.237	0.869	TIME	8.496	1.321	2.998	12.996
OLD	13.029	3.408	6.710	25.480	EDU	0.101	0.072	0.008	0.512

### 4.6 High-quality economic development index

Table 5 demonstrates the high-quality economic development index, Table 6 demonstrates the innovative development index, the coordinated development index, the green development index, the open development index, and the shared development index.

**Table 5:** High-quality economic development index.

Year	(1) East Region											
	Beijing	Tianjin	Hebie	Liaoning	Shanghai	Jiangsu	Zhejiang	Fujian	Shandong	Guangdong	Guangxi	Hainan
2000	0.238	0.117	0.062	0.089	0.108	0.066	0.051	0.079	0.074	0.079	0.081	0.106
2001	0.261	0.152	0.061	0.098	0.109	0.067	0.056	0.08	0.082	0.098	0.08	0.124
2002	0.247	0.134	0.064	0.106	0.111	0.069	0.06	0.074	0.095	0.098	0.079	0.129
2003	0.197	0.113	0.066	0.118	0.124	0.07	0.069	0.079	0.096	0.1	0.078	0.145
2004	0.155	0.112	0.064	0.141	0.127	0.071	0.07	0.069	0.103	0.099	0.091	0.126
2005	0.154	0.108	0.072	0.137	0.148	0.083	0.077	0.075	0.119	0.109	0.102	0.106
2006	0.151	0.108	0.077	0.152	0.175	0.086	0.087	0.081	0.092	0.115	0.103	0.097
2007	0.188	0.125	0.098	0.179	0.224	0.098	0.102	0.088	0.093	0.142	0.119	0.108
2008	0.19	0.131	0.114	0.209	0.228	0.107	0.118	0.1	0.1	0.155	0.127	0.118
2009	0.207	0.132	0.137	0.21	0.244	0.119	0.14	0.127	0.113	0.181	0.166	0.129
2010	0.268	0.149	0.151	0.23	0.265	0.131	0.168	0.144	0.138	0.243	0.182	0.143
2011	0.259	0.166	0.155	0.241	0.252	0.148	0.194	0.165	0.141	0.245	0.211	0.158
2012	0.273	0.185	0.174	0.26	0.283	0.17	0.204	0.185	0.177	0.263	0.229	0.175
2013	0.293	0.207	0.199	0.272	0.298	0.19	0.223	0.21	0.197	0.303	0.236	0.186
2014	0.339	0.277	0.217	0.278	0.316	0.208	0.226	0.218	0.194	0.293	0.258	0.196
2015	0.399	0.309	0.267	0.266	0.344	0.234	0.269	0.266	0.221	0.342	0.28	0.214
2016	0.432	0.315	0.276	0.291	0.406	0.259	0.271	0.283	0.233	0.353	0.296	0.225
2017	0.477	0.336	0.313	0.325	0.437	0.277	0.288	0.278	0.244	0.412	0.282	0.225
2018	0.486	0.362	0.347	0.344	0.433	0.321	0.331	0.338	0.268	0.493	0.333	0.241
2019	0.466	0.405	0.378	0.35	0.422	0.333	0.382	0.355	0.286	0.576	0.326	0.246
2020	0.455	0.334	0.334	0.348	0.416	0.297	0.352	0.324	0.268	0.466	0.33	0.248
Year	(2) West Region											
	Neimenggu	Chongqing	Sichuan	Guizhou	Yunnan	Xizang	Shhanxi	Gansu	Qinghai	Ningxia	Xinjiang	
2000	0.096	0.103	0.076	0.071	0.105	0.067	0.05	0.068	0.121	0.081	0.106	
2001	0.095	0.106	0.08	0.071	0.123	0.065	0.051	0.071	0.129	0.078	0.124	
2002	0.089	0.108	0.078	0.049	0.09	0.063	0.054	0.067	0.134	0.079	0.129	
2003	0.117	0.104	0.081	0.049	0.109	0.074	0.065	0.097	0.148	0.079	0.145	
2004	0.121	0.109	0.083	0.06	0.098	0.078	0.064	0.1	0.15	0.088	0.126	
2005	0.12	0.105	0.095	0.079	0.095	0.085	0.071	0.075	0.15	0.101	0.106	
2006	0.122	0.116	0.102	0.087	0.088	0.098	0.082	0.073	0.154	0.105	0.097	
2007	0.148	0.133	0.132	0.109	0.106	0.101	0.097	0.091	0.175	0.118	0.108	
2008	0.155	0.142	0.148	0.116	0.108	0.112	0.115	0.108	0.177	0.128	0.118	
2009	0.186	0.155	0.172	0.138	0.134	0.124	0.13	0.12	0.194	0.136	0.129	
2010	0.202	0.176	0.191	0.151	0.152	0.131	0.145	0.124	0.224	0.163	0.143	
2011	0.213	0.204	0.186	0.192	0.173	0.165	0.16	0.131	0.186	0.161	0.158	
2012	0.249	0.238	0.251	0.238	0.195	0.213	0.18	0.153	0.203	0.183	0.175	
2013	0.257	0.256	0.274	0.269	0.208	0.23	0.207	0.171	0.212	0.206	0.186	
2014	0.262	0.278	0.293	0.305	0.223	0.25	0.218	0.196	0.23	0.222	0.196	
2015	0.286	0.328	0.32	0.338	0.244	0.273	0.247	0.219	0.274	0.243	0.214	
2016	0.294	0.313	0.337	0.358	0.258	0.316	0.262	0.466	0.281	0.263	0.225	
2017	0.313	0.316	0.361	0.386	0.28	0.283	0.266	0.257	0.297	0.277	0.225	
2018	0.321	0.355	0.403	0.437	0.286	0.305	0.286	0.32	0.312	0.299	0.241	
2019	0.317	0.366	0.418	0.43	0.298	0.276	0.319	0.316	0.288	0.296	0.246	
2020	0.339	0.363	0.402	0.419	0.289	0.315	0.3	0.309	0.304	0.292	0.248	
Year	(3) Central Region											
	Shanxi	Jilin	Heilongjiang	Anhui	Jiangxi	Henan	Hubei	Hunan				
2000	0.07	0.066	0.056	0.051	0.066	0.067	0.077	0.082				
2001	0.08	0.069	0.059	0.052	0.067	0.065	0.078	0.082				
2002	0.079	0.063	0.062	0.053	0.069	0.08	0.076	0.078				
2003	0.092	0.083	0.067	0.062	0.07	0.068	0.077	0.072				
2004	0.092	0.087	0.065	0.059	0.071	0.067	0.077	0.077				
2005	0.101	0.088	0.085	0.067	0.083	0.074	0.093	0.089				
2006	0.114	0.088	0.09	0.084	0.086	0.078	0.095	0.096				
2007	0.149	0.11	0.116	0.123	0.098	0.1	0.117	0.113				
2008	0.175	0.121	0.127	0.134	0.107	0.106	0.127	0.122				
2009	0.2	0.131	0.165	0.154	0.119	0.131	0.141	0.145				
2010	0.218	0.14	0.174	0.166	0.131	0.142	0.168	0.165				
2011	0.222	0.153	0.187	0.219	0.148	0.151	0.197	0.184				
2012	0.235	0.16	0.224	0.243	0.17	0.192	0.22	0.216				
2013	0.252	0.172	0.251	0.264	0.19	0.204	0.25	0.234				
2014	0.262	0.18	0.273	0.279	0.208	0.225	0.273	0.255				
2015	0.29	0.2	0.333	0.305	0.234	0.257	0.315	0.282				
2016	0.301	0.224	0.342	0.318	0.259	0.273	0.338	0.304				
2017	0.293	0.233	0.385	0.336	0.277	0.292	0.342	0.316				
2018	0.35	0.292	0.396	0.37	0.321	0.348	0.387	0.344				
2019	0.336	0.306	0.403	0.406	0.333	0.348	0.41	0.359				
2020	0.334	0.258	0.385	0.389	0.297	0.323	0.385	0.349

**Table 6:** High-quality Economic Development Index (Specific index).

Region	Provinces and cities	Innovation Development Index			Coordinated Development Index			Green Development Index			Open Development Index			Shared Development Index		
		2000	01–19	2020	2000	01–19	2020	2000	01–19	2020	2000	01–19	2020	2000	01–19	2020
East	Beijing	0.522	…	0.501	0.643	…	0.586	0.445	…	0.544	0.574	…	0.463	0.391	…	0.550
East	Tianjin	0.126	…	0.295	0.569	…	0.587	0.444	…	0.477	0.395	…	0.394	0.169	…	0.285
East	Hebei	0.027	…	0.082	0.451	…	0.448	0.444	…	0.531	0.052	…	0.091	0.161	…	0.359
East	Liaoning	0.065	…	0.114	0.506	…	0.506	0.512	…	0.538	0.195	…	0.281	0.240	…	0.378
East	Shanghai	0.126	…	0.304	0.683	…	0.534	0.515	…	0.469	0.421	…	0.545	0.302	…	0.466
East	Jiangsu	0.059	…	0.273	0.502	…	0.514	0.463	…	0.521	0.304	…	0.272	0.178	…	0.399
East	Zhejiang	0.046	…	0.301	0.542	…	0.557	0.534	…	0.565	0.159	…	0.263	0.168	…	0.405
East	Fujian	0.053	…	0.153	0.539	…	0.510	0.535	…	0.586	0.366	…	0.196	0.114	…	0.293
East	Shandong	0.044	…	0.145	0.456	…	0.451	0.458	…	0.529	0.215	…	0.155	0.180	…	0.369
East	Guangdong	0.067	…	0.260	0.505	…	0.479	0.571	…	0.706	0.586	…	0.466	0.237	…	0.415
East	Guangxi	0.024	…	0.046	0.450	…	0.476	0.436	…	0.585	0.085	…	0.065	0.149	…	0.287
East	Hainan	0.004	…	0.040	0.565	…	0.575	0.555	…	0.517	0.271	…	0.219	0.088	…	0.287
Central	Shanxi	0.035	…	0.070	0.463	…	0.491	0.282	…	0.453	0.054	…	0.083	0.221	…	0.379
Central	Jilin	0.051	…	0.087	0.502	…	0.508	0.540	…	0.559	0.074	…	0.066	0.158	…	0.268
Central	Heilongjiang	0.048	…	0.090	0.491	…	0.529	0.602	…	0.658	0.083	…	0.126	0.142	…	0.336
Central	Anhui	0.033	…	0.146	0.454	…	0.471	0.486	…	0.522	0.046	…	0.201	0.098	…	0.315
Central	Jiangxi	0.042	…	0.089	0.500	…	0.485	0.450	…	0.609	0.047	…	0.194	0.124	…	0.276
Central	Henan	0.046	…	0.089	0.433	…	0.425	0.445	…	0.525	0.033	…	0.145	0.135	…	0.345
Central	Hubei	0.060	…	0.150	0.466	…	0.484	0.478	…	0.563	0.070	…	0.104	0.130	…	0.352
Central	Hunan	0.064	…	0.115	0.470	…	0.468	0.474	…	0.589	0.059	…	0.078	0.123	…	0.327
West	Neimeng	0.054	…	0.044	0.498	…	0.518	0.485	…	0.606	0.054	…	0.073	0.148	…	0.327
West	Chongqing	0.106	…	0.142	0.450	…	0.506	0.354	…	0.550	0.059	…	0.150	0.124	…	0.337
West	Sichuan	0.069	…	0.140	0.434	…	0.457	0.488	…	0.629	0.037	…	0.126	0.158	…	0.461
West	Guizhou	0.022	…	0.071	0.422	…	0.463	0.333	…	0.529	0.015	…	0.028	0.107	…	0.323
West	Yunnan	0.087	…	0.057	0.405	…	0.470	0.498	…	0.614	0.031	…	0.077	0.168	…	0.351
West	Xizang	0.005	…	0.020	0.476	…	0.553	0.508	…	0.394	0.017	…	0.038	0.070	…	0.472
West	Shaanxi	0.131	…	0.184	0.435	…	0.461	0.396	…	0.535	0.068	…	0.095	0.157	…	0.323
West	Gansu	0.060	…	0.101	0.431	…	0.466	0.365	…	0.487	0.041	…	0.128	0.134	…	0.333
West	Qinghai	0.021	…	0.105	0.515	…	0.585	0.387	…	0.417	0.062	…	0.012	0.141	…	0.394
West	Ningxia	0.040	…	0.067	0.543	…	0.582	0.293	…	0.399	0.122	…	0.043	0.136	…	0.312
West	Xinjiang	0.061	…	0.033	0.461	…	0.504	0.425	…	0.479	0.032	…	0.054	0.165	…	0.395

**Fig. 2:**
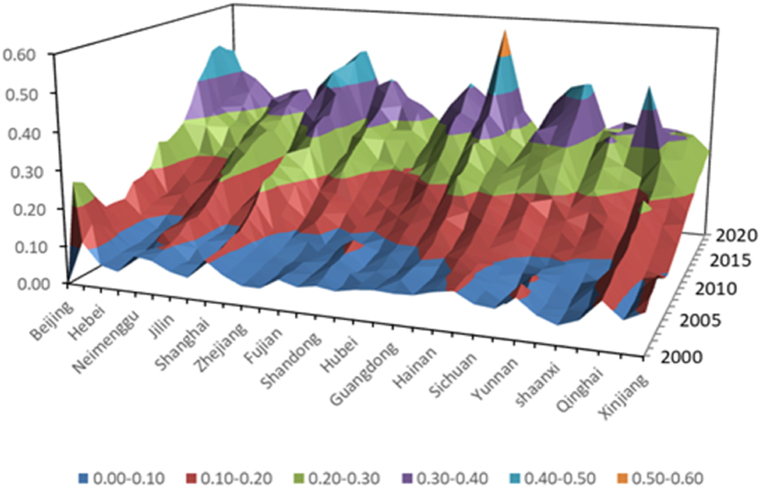
High-quality economic development index.

It can be seen from Table 5、Table 6, Fig. 2 that there are large development gaps in various aspects of China's 31 regions:

There is a significant gap in the innovation development index among different regions, with Beijing, Shanghai, and Zhejiang ranking in the top three. This is closely related to the fact that Beijing, Shanghai, and Zhejiang have always been at the forefront of innovation development. Beijing is a city with a relatively dense concentration of high-tech enterprises, which have injected new vitality into the economic innovation and development of Beijing. Shanghai is an international center for economic, financial, trade, shipping, and technological innovation, and its economic innovation and development are far ahead of many cities. Seven departments in Zhejiang jointly launched 20 measures to strengthen scientific and technological innovation to help stabilize and improve the economy. Hainan, Xinjiang, and Tibet rank in the bottom three, and there is still significant room for improvement in innovative development in these three regions' economies. Overall, the level of innovation and development in the eastern region is higher than that in the central region and higher than that in the western region.

There is a significant gap in the coordinated economic development index among different regions. Tianjin, Beijing, and Qinghai ranked in the top three in terms of coordinated development index, while Ningxia and Hainan ranked fourth and fifth in terms of coordinated economic development index. The western region has a good situation of coordinated economic development. Shandong, Hebei, and Henan provinces rank in the bottom three in terms of coordinated economic development, and there is still significant room for improvement in these three regions.

Guangdong, Heilongjiang, and Sichuan provinces rank among the top three in terms of green finance development among the 31 provinces. Although Heilongjiang province lags behind in terms of high-quality economic development, it has done a good job in green economy development. Sichuan Province has also done a good job in high-quality development of green economy due to its regional advantages. Qinghai, Ningxia, and Tibet rank third from bottom in terms of high-quality development of green economy. These three regions have poor economic development levels and have also done poorly in terms of high-quality green economy.

There is a significant gap in economic openness and development among different regions, with Shanghai, Guangdong, and Beijing having a relatively high level of economic openness and development, ranking in the top three. In 2020, the opening up and development index of the Shanghai region was 0.5452, ranking last in Qinghai Province. In 2020, the economic opening up and development index was only 0.0124. Overall, the western region is doing less in terms of economic openness and development. The eastern region, due to its geographical advantages and good economic development level, has overall done well in terms of opening up and development.

The overall economic sharing development of 31 provinces and municipalities is relatively high, with Beijing and Shanghai having the highest level of economic sharing development. Jiangxi Province and Jilin Province have done relatively poorly in terms of economic shared development, and the economic development of these two provinces is relatively weak. Overall, the central region's efforts in economic sharing and development are also weaker, which is in line with the overall level of economic development in various regions.

### 4.7 Carbon finance development index

**Fig. 3:**
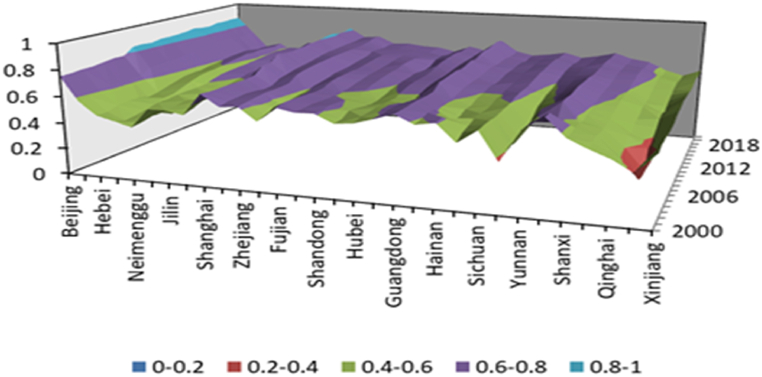
Carbon finance development index.

From Table 7, Fig. 3, it can be seen that the carbon finance development index has the following characteristics: There are significant differences in the development level of carbon finance among different regions. Beijing, Shanghai, Guangdong, Zhejiang, Jiangsu, Tianjin and other regions firmly occupy the first tier. The development level of carbon finance in the eastern region is relatively high, with some regions such as Hebei and Liaoning experiencing poor development among the 31 provinces, autonomous regions, and municipalities directly under the central government. The overall development level of carbon finance in the central region is at the intermediate level, while the development level of carbon finance in the western region is relatively backward.

**Table 7:** Carbon finance development index.

Year	(1) East Region											
	Beijing	Tianjin	Hebie	Liaoning	Shanghai	Jiangsu	Zhejiang	Fujian	Shandong	Guangdong	Guangxi	Hainan
2000	0.753	0.572	0.475	0.552	0.694	0.608	0.619	0.632	0.61	0.646	0.584	0.606
2001	0.754	0.582	0.477	0.575	0.686	0.624	0.628	0.643	0.604	0.647	0.598	0.612
2002	0.762	0.598	0.502	0.576	0.687	0.628	0.636	0.642	0.616	0.647	0.61	0.584
2003	0.763	0.621	0.509	0.585	0.687	0.631	0.643	0.636	0.61	0.653	0.611	0.537
2004	0.775	0.62	0.509	0.588	0.684	0.623	0.64	0.63	0.6	0.65	0.603	0.54
2005	0.786	0.622	0.487	0.59	0.683	0.613	0.64	0.623	0.577	0.646	0.602	0.565
2006	0.783	0.627	0.501	0.592	0.692	0.616	0.644	0.628	0.583	0.651	0.605	0.563
2007	0.839	0.672	0.522	0.614	0.746	0.651	0.687	0.653	0.608	0.697	0.619	0.587
2008	0.841	0.68	0.542	0.631	0.749	0.662	0.702	0.662	0.623	0.701	0.631	0.596
2009	0.834	0.683	0.563	0.638	0.797	0.677	0.709	0.663	0.635	0.708	0.638	0.61
2010	0.847	0.685	0.569	0.645	0.784	0.686	0.716	0.667	0.646	0.715	0.642	0.619
2011	0.847	0.695	0.57	0.647	0.781	0.693	0.719	0.664	0.653	0.706	0.643	0.607
2012	0.849	0.709	0.585	0.654	0.787	0.704	0.724	0.673	0.656	0.716	0.653	0.617
2013	0.857	0.72	0.591	0.662	0.791	0.715	0.726	0.681	0.673	0.732	0.665	0.631
2014	0.868	0.73	0.603	0.669	0.798	0.723	0.727	0.689	0.674	0.722	0.671	0.635
2015	0.856	0.737	0.611	0.676	0.8	0.726	0.728	0.693	0.675	0.747	0.663	0.638
2016	0.852	0.732	0.618	0.671	0.812	0.729	0.726	0.692	0.671	0.758	0.669	0.647
2017	0.867	0.732	0.62	0.67	0.816	0.733	0.731	0.694	0.674	0.759	0.673	0.646
2018	0.864	0.723	0.595	0.623	0.801	0.731	0.739	0.692	0.67	0.771	0.664	0.633
2019	0.869	0.72	0.604	0.627	0.801	0.738	0.757	0.699	0.676	0.778	0.667	0.645
2020	0.867	0.737	0.603	0.658	0.814	0.739	0.749	0.697	0.676	0.764	0.674	0.646
Year	(2) West Region											
	Neimenggu	Chongqing	Sichuan	Guizhou	Yunnan	Xizang	Shhanxi	Gansu	Qinghai	Ningxia	Xinjiang	
2000	0.406	0.48	0.578	0.373	0.606	0.699	0.569	0.506	0.453	0.376	0.516	
2001	0.416	0.523	0.583	0.392	0.599	0.699	0.572	0.52	0.452	0.314	0.543	
2002	0.435	0.54	0.586	0.419	0.597	0.603	0.578	0.534	0.423	0.256	0.535	
2003	0.434	0.561	0.58	0.404	0.595	0.61	0.581	0.527	0.411	0.237	0.547	
2004	0.397	0.572	0.585	0.415	0.632	0.695	0.564	0.522	0.404	0.25	0.529	
2005	0.408	0.565	0.594	0.445	0.574	0.697	0.584	0.528	0.456	0.374	0.532	
2006	0.39	0.571	0.598	0.449	0.58	0.691	0.59	0.537	0.445	0.375	0.533	
2007	0.422	0.592	0.617	0.491	0.595	0.7	0.605	0.555	0.474	0.408	0.557	
2008	0.445	0.596	0.626	0.537	0.62	0.706	0.615	0.572	0.484	0.435	0.565	
2009	0.484	0.612	0.629	0.549	0.622	0.701	0.624	0.59	0.493	0.471	0.569	
2010	0.51	0.617	0.63	0.572	0.633	0.7	0.627	0.59	0.527	0.483	0.586	
2011	0.5	0.625	0.644	0.579	0.636	0.699	0.627	0.598	0.519	0.452	0.584	
2012	0.515	0.635	0.657	0.593	0.642	0.691	0.634	0.604	0.524	0.483	0.58	
2013	0.545	0.651	0.668	0.612	0.657	0.69	0.645	0.619	0.545	0.5	0.576	
2014	0.557	0.648	0.673	0.625	0.666	0.695	0.654	0.63	0.56	0.519	0.565	
2015	0.573	0.65	0.682	0.643	0.677	0.695	0.661	0.644	0.576	0.539	0.571	
2016	0.569	0.665	0.686	0.647	0.672	0.704	0.659	0.647	0.587	0.544	0.583	
2017	0.567	0.663	0.69	0.653	0.674	0.706	0.664	0.648	0.589	0.552	0.591	
2018	0.556	0.659	0.678	0.643	0.665	0.696	0.656	0.633	0.571	0.54	0.579	
2019	0.551	0.662	0.684	0.642	0.665	0.701	0.655	0.632	0.565	0.534	0.578	
2020	0.565	0.662	0.684	0.647	0.67	0.702	0.659	0.64	0.576	0.538	0.587	
Year	(3) Central Region											
	Shanxi	Jilin	Heilongjiang	Anhui	Jiangxi	Henan	Hubei	Hunan				
2000	0.442	0.554	0.53	0.523	0.607	0.548	0.562	0.602				
2001	0.419	0.561	0.551	0.535	0.609	0.551	0.582	0.601				
2002	0.43	0.574	0.581	0.574	0.611	0.588	0.583	0.611				
2003	0.448	0.545	0.587	0.548	0.607	0.577	0.589	0.609				
2004	0.467	0.577	0.589	0.577	0.601	0.563	0.591	0.603				
2005	0.492	0.556	0.576	0.587	0.602	0.552	0.588	0.579				
2006	0.499	0.566	0.581	0.593	0.601	0.554	0.586	0.58				
2007	0.527	0.582	0.599	0.608	0.606	0.567	0.612	0.603				
2008	0.554	0.596	0.609	0.616	0.619	0.587	0.627	0.62				
2009	0.576	0.613	0.622	0.625	0.628	0.598	0.636	0.63				
2010	0.588	0.618	0.635	0.64	0.642	0.608	0.638	0.636				
2011	0.594	0.612	0.636	0.643	0.645	0.612	0.64	0.635				
2012	0.602	0.619	0.642	0.648	0.652	0.623	0.651	0.643				
2013	0.617	0.641	0.653	0.657	0.657	0.61	0.672	0.654				
2014	0.624	0.65	0.661	0.666	0.668	0.61	0.686	0.661				
2015	0.638	0.663	0.669	0.674	0.676	0.619	0.693	0.665				
2016	0.63	0.66	0.665	0.699	0.681	0.652	0.698	0.664				
2017	0.638	0.662	0.665	0.697	0.687	0.661	0.705	0.671				
2018	0.633	0.65	0.656	0.689	0.683	0.639	0.694	0.665				
2019	0.626	0.647	0.655	0.698	0.686	0.645	0.7	0.667				
2020	0.637	0.653	0.669	0.69	0.68	0.645	0.698	0.667

### 4.8 Analysis results of the impact of carbon finance development on high-quality economic development

From Table 8 It can be seen the fixed effects, random effects and system GMM models that carbon finance has a significant positive impact on high-quality economic development, and the impact coefficients are 0.422, 0.127 and 0.087, respectively. The empirical results are consistent with the hypothesis of H1 in this paper. In each model, the old-age dependency ratio has a significant positive impact on high-quality economic development, mainly because the family planning policy has played a huge role in reducing the child dependency ratio since the reform and opening up, coupled with the huge demographic dividend in the past three decades, China has achieved rapid economic growth, but the impact of aging on high-quality economic development cannot be ignored. In each model, the birth rate has a significant positive impact on high-quality economic development, which is consistent with theoretical analysis. The unemployment rate has a negative impact on high-quality economic development in the fixed effects and random effects models, which is consistent with theoretical analysis. In order to better achieve high-quality economic development, we need to promote employment. Government expenditure, transportation infrastructure construction, years of schooling and proportion of higher education population have positive effects on high-quality economic development in fixed effects and random effects models, while urbanization rate has positive effects on high-quality economic development in fixed effects and system GMM models, which is consistent with theoretical analysis. In order to better promote high-quality economic development, it is necessary to increase government spending, strengthen transport infrastructure, improve educational attainment, and promote new urbanization.

**Table 8:** Analysis results of the impact of carbon finance development on high-quality economic development.

Variable	Fixed effect	Random effect	System GMM	Variable	Fixed effect	Random effect	System GMM
L.HQED			0.673*** (22.840)				
CARBON	0.422*** (7.373)	0.127*** (6.045)	0.087** (2.370)	ZHF	0.011*** (3.852)	0.216*** (12.874)	0.019 (1.110)
OLD	0.007*** (8.722)	0.011*** (13.289)	0.002** (2.410)	INF	0.029*** (2.696)	0.022** (2.473)	−0.013 (−1.450)
CHILD	0.001 (0.792)	−0.001 (−0.034)	−0.009 (−1.930)	CHZ	0.001** (2.142)	0.003 (0.837)	0.001** (2.400)
BIRTH	0.005*** (3.415)	0.008*** (7.642)	0.002* (1.720)	TIME	0.021*** (2.856)	0.035*** (6.823)	0.006 (0.940)
SHIYE	−0.029*** (−7.975)	−0.021*** (−6.363)	−0.001 (−0.590)	EDU	0.563*** (6.372)	0.334*** (4.692)	−0.039 (−0.570)
C	−0.455*** (−6.928)	−0.544*** (−10.063)		R^2^	0.887	0.831	0.915

Note: L.HQED represents the lag term of high-quality economic development. ***, **, and * denote significance at the 1 %, 5 %, and 10 % levels, respectively. The same notation applies hereafter.

### 4.9 Path analysis

In the preceding study, it was determined that high-quality economic growth consists of five components: creative development, coordinated development, green development, open development, and shared development. It is unclear which factors carbon financing will influence. This study employs the spatial Durbin model to examine the effect of carbon finance on economic innovation development, economic coordinated development, economic green development, economic open development, and economic shared development in order to improve the analysis of the impact route of carbon finance development on high-quality economic growth.

Table 9 shows that the influence coefficient of carbon finance development on economic innovation development is 0.0583, but it is not significant. Various regions in China continue to promote innovation and development, but there is still much room for improvement in the degree of innovation and development, and the development of carbon finance has not directly affected economic innovation and development. The coefficient of influence on coordinated economic development is 0.0416, which is significant at the 5 % level. The influence coefficient on economic green development is 0.0618, which is significant at the 5 % level. The coefficient of influence on economic openness and development is 0.0095, but it is not significant, mainly because when constructing the economic openness development index, it is mainly measured from the perspective of investment openness and trade openness, and carbon finance development needs to be further improved in promoting investment openness and trade openness. The coefficient of influence on shared economic development is 0.0629, which is significant at the 5 % level. It can be seen from this result that carbon finance does not have a significant impact on the five aspects of high-quality economic development, but only has a positive effect on coordinated development, green development and shared development, which verifies H2 of this paper. These three aspects can be used as action paths to enhance the impact of carbon finance on high-quality economic development.

**Table 9:** Regression analysis results of the impact of carbon finance development on the path of high-quality economic development.

Variables	ID	CD	GD	OD	SD
L.HQED	0.5767*** (19.4500)	0.7482*** (26.8300)	0.5956*** (19.5700)	0.5592*** (17.7400)	0.5935*** (19.0700)
CF	0.05831 (1.6800)	0.0416** (2.2400)	0.0618** (2.1500)	0.0095 (1.1900)	0.0629** (2.2200)
ODR	0.0001 (0.0800)	0.0004 (1.0900)	0.0005 (1.0700)	0.0022 (1.6100)	0.0004 (0.7800)
CDR	−0.0005 (−1.0600)	−0.0001 (−0.5700)	0.0003 (1.0000)	0.0005 (0.5600)	−0.0001 (−0.1400)
PB	0.0019** (2.4100)	−0.0002 (−0.5300)	−0.0011* (−1.6900)	0.0024 (1.2900)	−0.0008 (1.2400)
RU	0.0031 (1.4800)	0.0009 (0.8500)	0.0017 (0.9900)	−0.0100** (−2.0900)	−0.0031* (−1.8400)
GOV	−0.0132 (−0.8200)	0.0062 (0.7100)	−0.0567*** (−4.3200)	0.0839** (2.300)	0.1226*** (8.0000)
INFT	−0.0089 (−1.0800)	0.0018 (0.4200)	−0.0175** (−2.5500)	−0.0292 (−1.5500)	−0.0026 (−0.3800)
URBZ	−0.0012*** (−3.3800)	0.0004** (2.3700)	0.0003 (1.3800)	−0.0008 (−1.0600)	0.0001 (0.4400)
EDUT	−0.0031 (−0.5100)	0.0056* (1.7400)	0.0001 (0.0001)	0.0084 (0.6200)	0.0015 (0.3100)
EDUB	0.2129*** (3.2800)	−0.0984** (−2.7900)	0.0150 (0.2800)	−0.1109 (−0.7500)	−0.0160 (−0.3000)
R^2^	0.8417	0.8321	0.7835	0.8352	0.8399

### 4.10 Heterogeneity analysis

#### 4.10.1 Regional heterogeneity analysis

There is an asymmetry in the economic growth of China's different regions, with the eastern region's degree of economic development being comparatively high. The findings of the calculations for the high-quality economic development index and the carbon finance development index indicate that both the economic high-quality development level and the carbon finance development level of the eastern area are quite high. For high-quality economic growth in China, it is essential that this research examine the variability of carbon financing in various locations. This article splits China's 31 provinces and cities into eastern, central, and western areas and examines the effect of carbon financing on high-quality economic growth in each region. Table 10 demonstrates the empirical findings.

**Table 10:** Results of the impact of carbon finance development on high-quality economic development by region.

Variables	Eastern Region	Central Region	Western Region
L.HQED	0.7056*** (16.3300)	0.5072*** (8.4000)	0.3607*** (6.4200)
CF	0.3655*** (3.6200)	0.0606 (1.4000)	−0.0465 (−0.7100)
ODR	0.0005 (0.5460)	−0.0001 (−0.0200)	0.0036** (2.3800)
CDR	−0.0012 (0.1870)	0.0005 (1.4200)	0.0002 (0.2300)
PB	0.0015 (1.0700)	−0.0003 (−0.3700)	0.0012 (0.7200)
RU	0.0066* (1.9400)	0.0011 (0.6900)	−0.0141*** (−2.6300)
GOV	0.1419** (2.1400)	−0.0354 (−0.7600)	0.0148 (0.5000)
INFT	−0.0310** (−2.4300)	0.0083 (1.3500)	0.0008 (−0.0500)
URBZ	0.0001 (−0.0600)	0.0017*** (3.0300)	0.0004 (0.2600)
EDUT	0.0158 (1.3000)	0.0005 (0.1100)	0.0016 (1.5300)
EDUB	0.0271 (−0.2600)	−0.0808 (−1.0800)	−0.1726 (−1.0600)
R^2^	0.9613	0.7493	0.9090

According to Table 10, there is a disparity between carbon financing and high-quality economic growth in various locations. It has a substantial beneficial influence on the quality economy growth in the eastern area. In the central and western areas, the growth of carbon financing has no appreciable effect on the quality of economic development. The possible reasons are as follows: First, high-quality economic development and carbon finance are concepts of sustainable development in recent years. In the economically developed eastern region, the provinces have a good economic foundation, are able to carry out high-quality economic transformation and have the ability to develop carbon finance. For example, Zhejiang Province in the eastern region has been rated as a green financial reform pilot zone by the state. The effect of carbon finance on high-quality economic development in these provinces and cities was reflected earlier. Second, due to the rapid economic development in the eastern region, the industrial structure has begun to transform into a service industry. Some manufacturing industries with high energy consumption and high pollution have been transferred to the central and western regions, but these industries are the main source of economic growth in the central and western regions. In the short term, the development of carbon finance cannot completely eliminate these industries. Third, the financial environment in the eastern region is better than that in the central and western regions. The eastern region pays more attention to the financial support of green environmental protection enterprises and high energy consumption enterprises, which makes the financial support of green environmental protection enterprises in the eastern region stronger. It can be seen that affecting the high-quality development of the economy through carbon finance requires the transformation of supporting industries, and the issue of heterogeneity should be considered.

#### 4.10.2 Temporal heterogeneity

Since the reforms and open-up of the Chinese economy, remarkable progress has been realized. High-quality growth was originally advocated during the 19th National Congress of the Chinese Communist Party in 2017, suggesting that China's economy has transitioned from a high-speed growth phase to a high-quality development phase. In order to determine if the influence of carbon financing on high-quality economic growth is consistent, this research uses 2017 as a dividing line to examine the temporal heterogeneity of carbon finance on high-quality economic development.

Table 11 demonstrates that the effect of carbon financing on high-quality economic growth is heterogeneous. Although the regression coefficients of carbon financing on high-quality economic growth are substantial based on the overall findings, there are contradictions. First, from the significance level of the regression coefficient, the regression coefficient from 2000 to 2016 was significant at the 10 % significance level, but from 2017 to 2020, the coefficient was significant at the 1 % significance level. This indicates that the influence of carbon financing on high-quality economic growth has grown increasingly substantial since China publicly adopted the idea of high-quality economic development. Second, from the regression results, the regression coefficient from 2000 to 2016 is 0.6390, indicating that every 1 % increase in carbon finance at this time can promote high-quality economic development by 0.64 %. The regression coefficient from 2017 to 2020 is 1.3471, indicating that every 1 % increase in carbon finance can promote high-quality economic development by 1.35 %, indicating that since 2017, the scale effect of carbon finance on high-quality economic development has been very significant, resulting in economies of scale. This result shows that large-scale development of high-quality economics can be achieved through the large-scale development of carbon finance.

**Table 11:** Results of the impact of carbon finance development on high-quality economic development by year.

Variables	2000–2016	2017–2020
L.HQED	0.8122*** (22.3300)	−0.1827*** (−2.4100)
CF	0.6390* (1.8600)	1.3471*** (4.7400)
ODR	0.0011* (1.6800)	0.0016 (1.1500)
CDR	0.0001 (0.1300)	−0.0046*** (−4.3100)
PB	0.0012 (1.3000)	−0.0014 (−0.7400)
RU	0.0002 (0.1100)	−0.0045 (−1.0700)
GOV	0.0569*** (3.1600)	−0.0952** (−2.5400)
INFT	−0.0081 (−0.8400)	−0.0441 (−1.6400)
URBZ	−0.0002 (−0.5800)	0.0088*** (2.9200)
EDUT	0.0079 (1.3800)	0.2692*** (2.7100)
EDUB	0.0019 (0.2900)	−2.0084** (−2.4100)
R^2^	0.9493	0.2310

## 5 Conclusions and policy recommendations

### 5.1 Conclusions

Prior literature offers a comprehensive analysis of the essence and metrics of high-quality economic development, serving as a foundation for this paper's high-quality economic development index system. Noteworthy research has been undertaken on carbon finance, its markets, and its impact on high-quality economic development. Yet, scholarly consensus on the construction of a high-quality economic development index system remains elusive. A majority of scholars have focused on general metrics for high-quality economic development, with only a handful delving into specific indicators such as innovative, coordinated, green, open, and shared development. While abundant theoretical work exists on carbon finance, there is a scarcity of literature developing and computing a carbon finance index system. In a bid to bolster research in high-quality economic development, this study meticulously measures the economic indices of innovative, coordinated, green, open, and shared development using the entropy weight method. Additionally, this paper broadens the scope of carbon finance research by constructing and computing a carbon finance index system. Findings reveal disparities in the high-quality economic development index across regions and years, with the overarching trend indicating a positive correlation between the index and regional economic development levels. The innovation development indices for Beijing, Shanghai, and Zhejiang are paramount, with the eastern region outperforming both the central and western regions. Marked variations exist in the coordinated economic development index among regions, with Tianjin, Beijing, and Qinghai leading the ranks. Guangdong, Heilongjiang, and Sichuan excel in green finance development. Economic openness and development indices display stark regional contrasts, with Shanghai, Guangdong, and Beijing as frontrunners. Owing to its geographical benefits and superior economic progression, the eastern region excels in openness and development. Most regions have commendable scores in economic shared development, with Beijing and Shanghai at the zenith. As expected, the central region's performance in shared economic development is somewhat lackluster, aligning with its overall economic standing. Concurrently, regional disparities manifest in carbon finance development levels, with the eastern region leading, the central region holding an intermediate position, and the western region lagging behind.

This study employs both static and dynamic panel models to empirically evaluate the influence of carbon finance on China's high-quality economic development. A detailed analysis elucidates the specific impact pathways, regional disparities, and temporal heterogeneities of carbon finance on such development. Results indicate that carbon finance considerably augments high-quality economic development. Interestingly, carbon finance does not exert significant influence across all five dimensions of high-quality economic development but predominantly enhances coordinated, green, and shared development. Regional disparities emerge in the correlation between carbon finance and high-quality economic development. For the eastern region, a pronounced positive influence on high-quality economic development is evident. Conversely, in the central and western regions, carbon finance development appears not to significantly affect high-quality economic development. Notably, around the year 2017, the impact of carbon finance on high-quality economic development exhibits marked heterogeneity.

This study enhances understanding in the realms of high-quality economic development and carbon finance evolution, offering researchers a novel framework for the selection of indicators to compute both the high-quality economic development index and the carbon finance development index. Concurrently, it furnishes a benchmark for regions to comprehend more deeply their economic development quality and the maturity of their carbon finance. Moreover, this investigation augments the empirical scrutiny concerning carbon finance's influence on high-quality economic development. It broadens the exploration of impact pathways and the inherent heterogeneities of carbon finance on economic progress, facilitating a more profound understanding of its operational routes, regional disparities, and temporal variations. Nevertheless, refinements can still be made in the computation of these indices. Data for 2021 and 2022 remain unavailable, with computations currently spanning only 2000–2020, thus excluding the indices for the most recent two years.

### 5.2 Policy implications

Empirical analyses reveal that factors such as carbon finance, government expenditure, transportation infrastructure development, urbanization rate, and educational advancements exert a positive influence on high-quality economic development. To further foster such development, several initiatives can be considered:

To advance high-quality economic development, it is imperative to cultivate an environment conducive to the growth of carbon finance and leverage its role in driving economic progress. Governments have an essential role in facilitating the evolution of carbon finance through diverse strategies. Born in the context of global climate concerns, carbon finance has a pronounced reliance on policy support. Hence, governmental influence becomes a critical determinant for its progression. Governments ought to vigorously back financial entities engaging in carbon finance, offering both fiscal and legal assistance for carbon-related financial endeavors. By extending support in the realms of taxation and subsidies, the state can mitigate potential policy risks. Concurrently, there is a need to enhance foundational infrastructures and promulgate policies that incentivize energy conservation and emission reduction efforts, thus catalyzing the eco-friendly transformation of enterprises. Furthermore, promoting low-carbon corporations to secure financing via securities markets can help expand enterprise horizons and foster a favorable business milieu. The enlargement of the carbon finance participant base is essential, with an emphasis on drawing more low-carbon industries. Such an approach would initiate a domino effect, starting with the power generation sector and permeating through various industries, thus broadening the carbon trading market's scope. Considering carbon finance's novelty, its intricate trading procedures require an acclimatization period for stakeholders, including governments, enterprises, and investors. A surge in trading opportunities will likely galvanize more corporations' active engagement. Emphasis should also be placed on devising innovative carbon financial instruments and services. This includes the exploration of groundbreaking carbon financial products such as carbon insurance, carbon funds, forward futures related to carbon, carbon securities, and other innovative derivatives, all aimed at amplifying the liquidity of the carbon trading marketplace.

The implications of aging are paramount. In phases characterized by advanced aging, a declining child dependency ratio foretells heightened aging challenges in the future. Consequently, shifts in the child dependency ratio considerably influence household savings and consumption decisions. The evidence suggests that as China's aging intensifies, the perturbations in the population's age structure have an augmented impact on high-quality economic development. Hence, during profound aging periods, China must bolster its social security framework, inaugurate financial markets and other elder-centric systems. This would attenuate the influences of elderly-associated burdens on households' judicious investments and precautionary savings. Thus, harmonizing the interplay between demographic transitions and high-quality economic development becomes essential.

Maximize the government's function. While emphasizing the market's primacy in steering high-quality economic development, it remains essential for the government to exert its regulatory roles, specifically in rectifying market inefficiencies. By doing so, the government can operate with enhanced efficiency and further propel high-quality economic development. Moreover, given the varied developmental statuses across regions, policies should be tailored to each region's unique circumstances. In extending policy support, it's crucial to avoid excessive biases, as these can impede a region's optimal economic advancement.

Prioritizing the development of transport infrastructure is vital for economic progression. It's imperative to champion the principles of sustainable transportation, meticulously aligning with natural landscapes and optimizing local conditions. Accelerating transportation infrastructure projects not only ensures unimpeded traffic and logistical flows but also establishes crucial transportation channels and enhances local connectivity. Emphasis should be placed on enhancing modern infrastructure, elevating the standards of transport services, supporting rural revitalization, fostering regional development synergy, and spearheading technological advancements in transportation. This approach aims to establish a comprehensive national three-dimensional transport grid, intensifying effective investments, and hastening the formation of an integrated, open transport market.

Advancing the development of new urbanization strategies, there is an emphasis on county towns as pivotal entities. The focus remains on harmonized growth between urban and rural areas and the integration of human, production, water, and land resources. This progression includes speeding up the urbanization process of rural migrants and amplifying the unification of foundational services at the county level, such as education, healthcare, and public cultural amenities. Enhancements in urban governance are paramount, coupled with the promotion of cultural values. Rural revitalization is essential, with an objective to ensure that the rewards of urbanization are equitably distributed among all urban and rural inhabitants.

Elevating educational standards involves the roll-out of comprehensive educational expansion initiatives, enhancing the infrastructure of educational institutions, bolstering teacher development, and advancing the general accessibility of education across all tiers. Efforts must be redoubled to sustain gains made in reducing compulsory education dropouts. A sustainable framework is required to magnify the achievements in education-driven poverty alleviation, steering compulsory education towards a trajectory of high-quality, equitable growth.

It is vital to solidify and expand the reach of senior high school education, synchronizing the growth of secondary vocational and regular high school education. An exploration into incentives encouraging junior high school graduates to pursue senior high school education is underway. The qualitative development of higher education should be promoted, pushing for wider accessibility. The equitable distribution of educational resources across regions, coupled with the formation of a proficient, quality-centric teaching force, is imperative to achieving educational fairness.

## Data availability statement

The original contributions presented in the study are included in the article/Supplementary Material, further inquiries can be directed to the corresponding author.

## Publisher's note

All claims expressed in this article are solely those of the authors and do not necessarily represent those of their affiliated organizations, or those of the publisher, the editors and the reviewers. Any product that may be evaluated in this article, or claim that may be made by its manufacturer, is not guaranteed or endorsed by the publisher.

## CRediT authorship contribution statement

**Lili Jiang:** Writing – original draft, Funding acquisition. **Huawei Niu:** Writing – review & editing. **Yufan Ru:** Methodology, Data curation, Conceptualization. **Aihua Tong:** Methodology, Formal analysis. **Yifeng Wang:** Writing – original draft, Supervision.

## Declaration of competing interest

The authors declare that they have no known competing financial interests or personal relationships that could have appeared to influence the work reported in this paper.
